# Risk‐Resilience Feedback to Assure Critical Societal Functions

**DOI:** 10.1111/risa.70197

**Published:** 2026-02-19

**Authors:** Samrat Chatterjee, Auroop Ganguly, Dennis Thomas, Lauren Wind

**Affiliations:** ^1^ Data Sciences and Machine Intelligence Group Pacific Northwest National Laboratory Richland Washington USA; ^2^ Sustainability and Data Sciences Laboratory Northeastern University Boston Massachusetts USA; ^3^ Computational Biology Group Pacific Northwest National Laboratory Richland Washington USA; ^4^ Systems Planning and Analysis, Inc. Alexandria Virginia USA

**Keywords:** compound and cascading risks, disaster policy, infrastructure resilience, natural‐built‐human systems, network science

## Abstract

Increase in compound natural and man‐made extreme events, and growing interconnectedness of critical societal functions emphasize further understanding of interactions between elements of risk and resilience. While conceptual frameworks have mapped quantified risks to the failure of critical societal functions, feedback from failure and recovery pathways to management of risk has not been systematically considered yet. In this brief perspective, we revisit the risk‐resilience feedback paradigm in the context of connected critical societal functions and discuss its role in advancing science‐informed disaster policy development. Specifically, we focus on connecting robustness and recovery elements of resilience to the management of risk elements, including threat, vulnerability, and consequence. Furthermore, we briefly describe a notional networked supply chain system example to illustrate how robustness and recovery measures can inform elements of risk. Understanding interactions between elements of risk and resilience is not a fully solved problem. Extending these concepts in the context of connected natural‐built‐human systems subject to compound and cascading risks is an open challenge. This discussion represents steps toward addressing these challenges.

## 1 Introduction

Communities around the globe are increasingly being stressed by two mutually strengthening challenges (National Research Council 2012; Helbing 2013; Kruczkiewicz et al. 2021; National Academies of Sciences, Engineering, and Medicine 2022; U.S. Cybersecurity and Infrastructure Security Agency 2024). First, compound natural and man‐made extreme events appear to be intensifying in magnitude, duration, and frequency, growing more correlated in space and time, and leading to larger losses of lives, property, and disruptions in business and lifestyles. Second, critical societal functions and communities across scales are growing more interconnected, leading to higher probabilities of cascading failures, which in turn are exacerbated by growing inequity and lack of adequate governance structures. The nature of these challenges has motivated the research community to think beyond probabilistic risk assessment into what has been called a resilience paradigm (Linkov et al. 2014). This new paradigm attempts to enhance and complement traditional risk assessments, which are based on threat, vulnerability, and consequence (Willis 2007; U.S. Department of Homeland Security 2010) applied to system components, with a better understanding of the inherent fragility (Rinaldi et al. 2001; Vespignani 2010; Buldyrev et al. 2010; Alderson et al. 2015; Duan et al. 2019; Eisenberg et al. 2019), stability (Gao et al. 2016), and the recovery potential (Bhatia et al. 2015; Yadav et al. 2020; Smith et al. 2020; Danziger and Barabási 2022) of highly interdependent systems and the critical societal functions they enable.

While the proposed resilience paradigm (Linkov et al. 2014) and the extant literature (Rinaldi et al. 2001; Haimes 2009; Vespignani 2010; Buldyrev et al. 2010; Alderson et al. 2015; Bhatia et al. 2015; Gao et al. 2016; Aven 2019; Duan et al. 2019; Eisenberg et al. 2019; Yadav et al. 2020; Smith et al. 2020; Chatterjee et al. 2021; Danziger and Barabási 2022) take important steps towards integrating traditional risk approaches with failure and recovery pathways, a gap remains in the state‐of‐the‐art. *Fragility*, including graceful degradation (where key functionality is sought to be maintained despite failures of components or subsystems), and *recovery*, including antifragility (where lessons learned from failures are used to recover the system to a better state than before), are not just informed by risks, but in turn influence the management of risks, thus leading to a feedback loop. However, the extant literature and state‐of‐the‐art practices are not yet able to appropriately account for this feedback or adapt it for developing practical solutions, such as for cyber‐physical critical infrastructure and supply chain systems. This gap has haunted communities and nations across disasters ranging from the tsunamis in Japan and in the Indian Ocean to hurricanes and wildfires in the United States, flooding in Asia and Europe, and even the COVID‐19 pandemic.

This brief perspective contributes to this ongoing discussion by highlighting further specific connections from robustness and recovery elements of resilience to management of risk, including threat, vulnerability, and consequence elements. In Section 2, these resilience‐to‐risk connections are discussed for both natural and man‐made failures and further represented through a notional networked supply chain system example with an eye toward quantification and broader adoption for policy insights. Section 3 contains concluding remarks.

## 2 Risk‐Resilience Feedback

Probabilistic risk analysis typically relies on threat, vulnerability, and consequence, while resilience tends to emphasize stability, robustness, and recovery. Conceptual frameworks have attempted to unify risk and resilience paradigms by equating quantified risks to plausible degradation of critical societal functions (Linkov et al. 2014). However, the feedback from anticipated failure and restoration pathways to risk management has not been systematically considered yet by scientists or practitioners. There is a growing body of recent literature that conceptually outlines the role of systems and network‐based approaches for further exploring connections between elements of *risk* and *resilience* (Rinaldi et al. 2001; Haimes 2009; Vespignani 2010; Buldyrev et al. 2010; Alderson et al. 2015; Bhatia et al. 2015; Gao et al. 2016; Aven 2019; Duan et al. 2019; Eisenberg et al. 2019; Smith et al. 2020; Yadav et al. 2020; Danziger and Barabási 2022). A systems approach to resilience and policy challenges has been proposed to further understand complex risks (Pescaroli and Alexander 2018; Hynes et al. 2020). In the context of complex extreme weather risks, a risk assessment framework may include interactions among risk drivers and multiple risks emerging from impacts and responses (Simpson et al. 2021). Recently, a systems‐based approach for combined risk and resilience stress testing was proposed with applicability to critical infrastructure (Linkov et al. 2022). This stress testing approach outlines multiple methods with varying data needs and modeling complexity that may yield a spectrum of possible analytic results. While systems and network‐based approaches have been proposed to conceptually understand and quantify risk and resilience elements, these methods are not fully fleshed out yet to be widely adopted for infrastructure policy and management. Furthermore, even as extremes have tended to intensify owing to drivers ranging from geopolitical and societal tensions to environmental change, their impacts have been magnified by the growing interdependence of natural, human‐engineered, and social systems, leading to the possibility of cascading failures. However, a near‐exclusive focus on component‐level robustness and/or on functions defined based on system attributes has introduced additional challenges (Linkov et al. 2022). What has been missing in practice and research is a due consideration of critical stakeholder functions. These dual gaps in the literature and best practice stymy effective design of policy, development of financial incentives, and community action. Advancing science‐informed disaster policy requires revisiting the risk‐resilience feedback paradigm in the context of national critical functions (U.S. Cybersecurity and Infrastructure Security Agency 2024; European Commission 2024; Secor et al. 2024).

**FIGURE 1:**
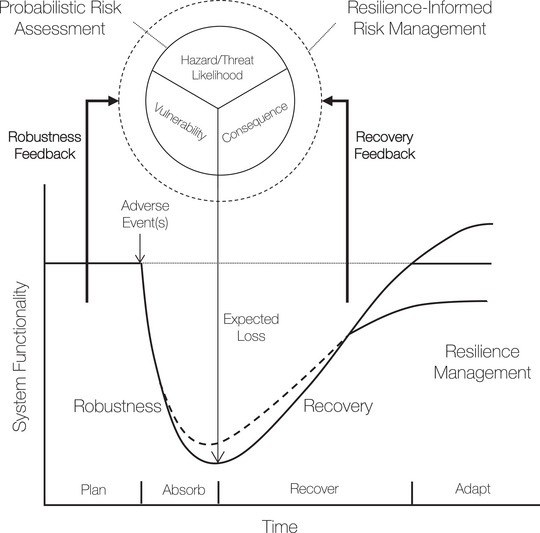
Illustration of robustness and recovery feedback to resilience‐informed risk management.

Given this backdrop, an effective and resilient science‐informed disaster policy requires further development of an analytical framing paradigm that includes effects from hazard/threat (representing both natural and intentional adverse events) failure and recovery patterns to broader risk management. Recently, risk has been interpreted as overall reduction in a system's functionality following a disruption (often expressed using expected loss) and resilience encapsulates time‐varying impacts of disruptive events (National Research Council 2012; Linkov et al. 2014). Motivated by Linkov et al. (2014) and considering compound and cascading risks, such a complementary risk‐resilience framing may be advanced further by introducing robustness and recovery feedback as part of resilience management to broader resilience‐informed risk management (see Figure 1). Effective robustness and recovery measures may eventually contribute to risk management by reducing hazard/threat event likelihood, vulnerability, and/or consequences. Such risk‐resilience feedback may ensure that a system exhibits graceful degradation and efficient recovery while maintaining mission‐critical functions, especially under compromise. This may be important due to the increasing compound nature of disruptions, interdependent system design and operations with coupling of network topologies and dynamics, and multi‐criteria, multi‐objective stakeholder priorities and decisions. Thus, a broader resilience‐informed risk management must include feedback from robustness and recovery phases to inform systemic risk policy and governance.

In Figure 1, risk quantification includes analyzing threat, vulnerability, and consequence of disruptive event(s), often via probabilistic methods. Risk quantification may inform robustness, especially the concept of graceful degradation—where a system (e.g., supply chain or critical infrastructure network) fails while still maintaining mission‐critical functions. The dashed degradation curve following the event represents a failure that is relatively more robust compared to the solid curve. Resilience management focuses on efficient recovery—where a system's functionality is restored rapidly under resource constraints and may even surpass prior levels through adaptation mechanisms. Recovery curves may also exhibit post‐event functionality, where the dashed curve represents recovery that is relatively more efficient compared to the solid curve, leading to final states of system functionality representing sub‐optimality (solid line below prior level), optimality (solid line at prior level), and anti‐fragility (solid line beyond prior level). Thus, robustness and recovery mechanisms may generate feedback to a broader resilience‐informed risk management.

**FIGURE 2:**
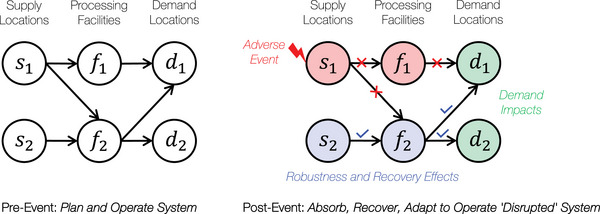
Resilience‐informed risk management concept in a notional networked supply chain system.

Figure 2 presents a notional networked supply chain system example to further elucidate the resilience‐informed risk management concept with robustness and recovery feedback. Consider *pre‐event* system state functionality involving planning and system operations with supply locations $\left\{s_{1},s_{2}\right\}$, processing facilities $\left\{f_{1},f_{2}\right\}$, and demand locations $\left\{d_{1},d_{2}\right\}$. Assuming adequate security and robustness measures are present at supply location $s_{2}$, adversarial threats may shift toward supply location $s_{1}.\;$ Following such an adverse event impacting supply location $s_{1}$, *post‐event* system state functionality may involve disruptive impacts that cascade to processing facility $f_{1}$ with no commodity transfer from $s_{1}$ and further from $f_{1}$. Such a disruption may eventually impact demand at locations $d_{1}$ and $d_{2}$. However, robustness and recovery effects due to reliance on backup resources at a secure supply location $s_{2}$ and increased capacity at processing facility $f_{2}$ may lead to reduced vulnerability of operational disruption at these locations, resulting in minimal systemic demand impacts. Thus, effective robustness and recovery measures can influence system risk elements by reducing threat likelihood, vulnerability, and consequences due to an adverse threat event. Further, in the case of a natural hazard event impacting supply location $s_{1}$, effective robustness and recovery measures at supply location $s_{2}$ and processing facility $f_{2}$ may lead to reduced vulnerability of operational disruption and consequences due to systemic demand impacts.

The need for revisiting the risk‐resilience paradigm and motivating novel solutions can be further illustrated through recent adverse events. Consider failure and recovery patterns following hurricanes Katrina, Sandy, Harvey, and Maria, among the top few devastating Atlantic hurricanes. During Katrina, failure of the flood protection systems around New Orleans largely contributed to loss of lives and destruction of transportation and communication systems, thereby cutting off access to basic supplies. A drastic failure, coupled with a gradual response and recovery process, collectively contributed to risk management challenges. In the case of Sandy, lessons learned from Katrina led to pre‐planning and preparation efforts ahead of time and resulted in relatively rapid restoration of power and building repairs in New York City, thereby limiting losses. During Harvey, Houston's aging and inadequate stormwater drainage systems, coupled with rapid population growth and lack of zoning regulations, led to significant flooding impacts. Evacuation planning and preparedness contributed positively to the recovery and restoration process. However, the combined effect of Harvey and the ongoing COVID‐19 pandemic resulted in income and employment losses. Finally, Hurricane Maria caused catastrophic destruction in Puerto Rico in part due to its aging infrastructure and inadequate maintenance of electric grid and water systems, compounded further by resource constraints and a slow recovery process. In South Asia, Cyclone Fani—categorized as “extremely severe” with an average wind speed of 120 mph–impacted the relatively poor state of Odisha on India's eastern coast. However, disaster preparation and timely evacuation planning and response to move about 1.4 million people to more than 900 cyclone shelters resulted in only about 40 casualties. Switching focus to severe winter storms, the power crisis in Texas led to statewide outages as well as food and water shortages, largely due to lack of winterization of power equipment. Furthermore, the Colonial pipeline ransomware attack highlighted the susceptibility of cyber‐physical infrastructure systems to compromise and the risks due to connections between information and operational technologies. Societal impacts due to pipeline shutdown for about a week ranged from fuel shortages to flight schedule changes. These examples further illustrate complexity in the ways in which hazard/threat failure and recovery patterns affect compound and cascading risk management.

## 3 Concluding Remarks

Understanding interactions between elements of risk (i.e., threat, vulnerability, and consequence) and resilience (i.e., planning, absorption, recovery, and adaptation) is not a fully solved problem. Moreover, extending these concepts in the context of connected natural‐built‐human systems subject to compound and cascading risks is an open challenge for researchers and a grand challenge for society. Elements of resilience, specifically failure and recovery pathways, may inform elements of risk and its management. The purpose of this brief perspective is to motivate the risk science community to think further about the proposed conceptual resilience‐informed risk management framing and develop new methods and validation strategies that quantify and evaluate the risk‐resilience feedback.

## Funding

This study was supported by the US Cybersecurity and Infrastructure Security Agency's National Risk Management Center program–National Infrastructure Simulation and Analysis Center (S.C., D.T., A.G.) and the US Department of Defense Strategic Environmental Research and Development Program (SERDP) grant RC20–1183 (A.G.).
